# Peripodial adherens junctions regulate Ajuba-Yorkie signaling to preserve fly eye morphology

**DOI:** 10.1242/bio.059579

**Published:** 2023-03-27

**Authors:** Dana F. DeSantis, Scott J. Neal, Qingxiang Zhou, Francesca Pignoni

**Affiliations:** ^1^ Department of Neuroscience and Physiology, Upstate Medical University, 505 Irving Avenue, NRB 4610, Syracuse, NY 13210, USA; ^2^ Department of Ophthalmology and Visual Sciences, Upstate Medical University, 505 Irving Avenue, NRB 4610, Syracuse, NY 13210, USA; ^3^ Department of Biochemistry and Molecular Biology, Department of Cell and Developmental Biology, Upstate Medical University, 505 Irving Avenue, NRB 4610, Syracuse, NY 13210, USA

**Keywords:** Epithelial morphology, Junctions, Yki, YAP, ECM, Tension, Laminin

## Abstract

The *Drosophila* eye develops from the larval eye disc, a flattened vesicle comprised of continuous retinal and peripodial epithelia (PE). The PE is an epithelium that plays a supporting role in retinal neurogenesis, but gives rise to cuticle in the adult. We report here that the PE is also necessary to preserve the morphology of the retinal epithelium. Depletion of the adherens junction (AJ) components β-Catenin (β-Cat), DE-Cadherin or α-Catenin from the PE leads to altered disc morphology, characterized by retinal displacement (RDis); so too does loss of the Ajuba protein Jub, an AJ-associated regulator of the transcriptional coactivator Yorkie (Yki). Restoring AJs or overexpressing Yki in β-Cat deficient PE results in suppression of RDis. Additional suppressors of AJ-dependent RDis include knockdown of Rho kinase (Rok) and Dystrophin (Dys). Furthermore, knockdown of βPS integrin (Mys) from the PE results in RDis, while overexpression of Mys can suppress RDis induced by the loss of β-Cat. We thus propose that AJ-Jub-Yki signaling in PE cells regulates PE cell contractile properties and/or attachment to the extracellular matrix to promote normal eye disc morphology.

## Teaser

**Summary:** Adherens junctions in the peripodial epithelium of the *Drosophila* eye disc promote normal morphology of the retinal epithelium and fly eye via a Jub-Wts-Yki-ECM signaling axis.

## INTRODUCTION

The *Drosophila* compound eye has long been used as a model for the study of epithelial patterning and organogenesis (reviewed in Kumar, 2018; Wolff and Ready, 1993; Treisman, 2013). It develops from a simple neuro-epithelial vesicle, called the eye imaginal disc (eye disc). Early in larval development, eye discs are partitioned into two morphologically distinct domains, the columnar retinal epithelium and the overlaying squamous peripodial epithelium (PE); cuboidal PE cells are found at the margin of these domains (Atkins and Mardon, 2009; Singh et al., 2012; Weasner and Kumar, 2022). Although the PE does not contribute to any portion of the fly eye, it is essential for multiple aspects of retinal development. Compromising the PE, through ablation or via genetic manipulations, results in reduced retinal cell proliferation and survival, arrest of retinal neurogenesis and abnormal ommatidia (single eyes) (Cho et al., 2000; Gibson et al., 2002; Gibson and Schubiger, 2000; 2001; Atkins and Mardon, 2009; Baker et al., 2018; McClure and Schubiger, 2005; Ramirez-Weber and Kornberg, 2000; Stultz et al., 2012; Won et al., 2015). In this study, we present evidence that adherens junctions (AJs) in the PE are required to maintain normal retinal epithelium morphology during larval development and to generate the precise architecture of the *Drosophila* compound eye.

AJs are highly conserved throughout Metazoa and are composed primarily of α-Catenin (α-Cat), β-Catenin (β-Cat), and E-Cadherin (E-Cad) (reviewed in Harris and Tepass, 2010; Gates and Peifer, 2005). The respective *Drosophila* orthologs are α-Cat, Armadillo (Arm) and DE-Cadherin (DE-Cad), the latter encoded by the *shotgun* (*shg*) locus. β-Cat and E-Cad directly interact and are mutually interdependent for their stability and trafficking to the plasma membrane (Hinck et al., 1994; Chen et al., 1999; Huber and Weis, 2001), where homotypic binding of E-Cad from neighboring cells mediates cell–cell adhesion. In the cytosol, Arm/β-Cat also binds α-Cat, which connects AJs to the actin cytoskeleton, and thus provides a means by which mechanical forces may be distributed across an epithelium (Harris and Tepass, 2010; Arnold et al., 2017; Heller and Fuchs, 2015; Wickstrom and Niessen, 2018).

AJs have also been recently identified as signaling platforms from which the Hippo signaling cascade may be modulated (Kim et al., 2011; Rauskolb et al., 2019; 2014; Sarpal et al., 2019; Schlegelmilch et al., 2011; Silvis et al., 2011). In this cascade, Hippo (Hpo) kinase (vertebrate STK3/MST1) phosphorylates the kinase Warts (Wts; vertebrate LATS1/2), which subsequently phosphorylates the transcriptional co-activator Yki (vertebrate YAP1/WWTR1), leading to its sequestration by the cytoplasmic anchor protein 14-3-3 (Huang et al., 2005; Pan, 2010). One means by which AJs can regulate Yki activity is via Ajuba LIM domain proteins (*Drosophila* Jub). These adaptor proteins conditionally localize to AJs and link cell–cell adhesion to diverse intracellular signaling pathways and their downstream transcriptional regulators, including the Hpo-Yki axis (Das Thakur et al., 2010; Jagannathan et al., 2016; Jia et al., 2020; Schleicher and Schramek, 2021). In the *Drosophila* wing disc, AJ-associated Jub binds to Wts and sequesters it, impeding its negative regulation of Yki (Rauskolb et al., 2019; 2014; Sarpal et al., 2019). This mechanism has been shown to locally regulate cell proliferation in different regions of the wing disc (Pan et al., 2018).

In this manuscript, we report that AJs in the eye disc PE promote normal retinal epithelium and eye morphology via Jub-Yki signaling. Compromising AJs in the PE results in a retinal displacement (RDis) phenotype, whereby a portion of the developing retinal epithelium comes to lie on the side of the disc normally occupied entirely by the PE, with ultimate consequences on adult eye formation. We provide evidence that altered Yki activity underlies RDis through a process that involves the modulation of tension and/or attachment to the extracellular matrix, and that is independent of Arm/β-Cat nuclear function.

## RESULTS

### Loss of Armadillo in the eye disc PE results in abnormal eye development

**Fig. 1.**
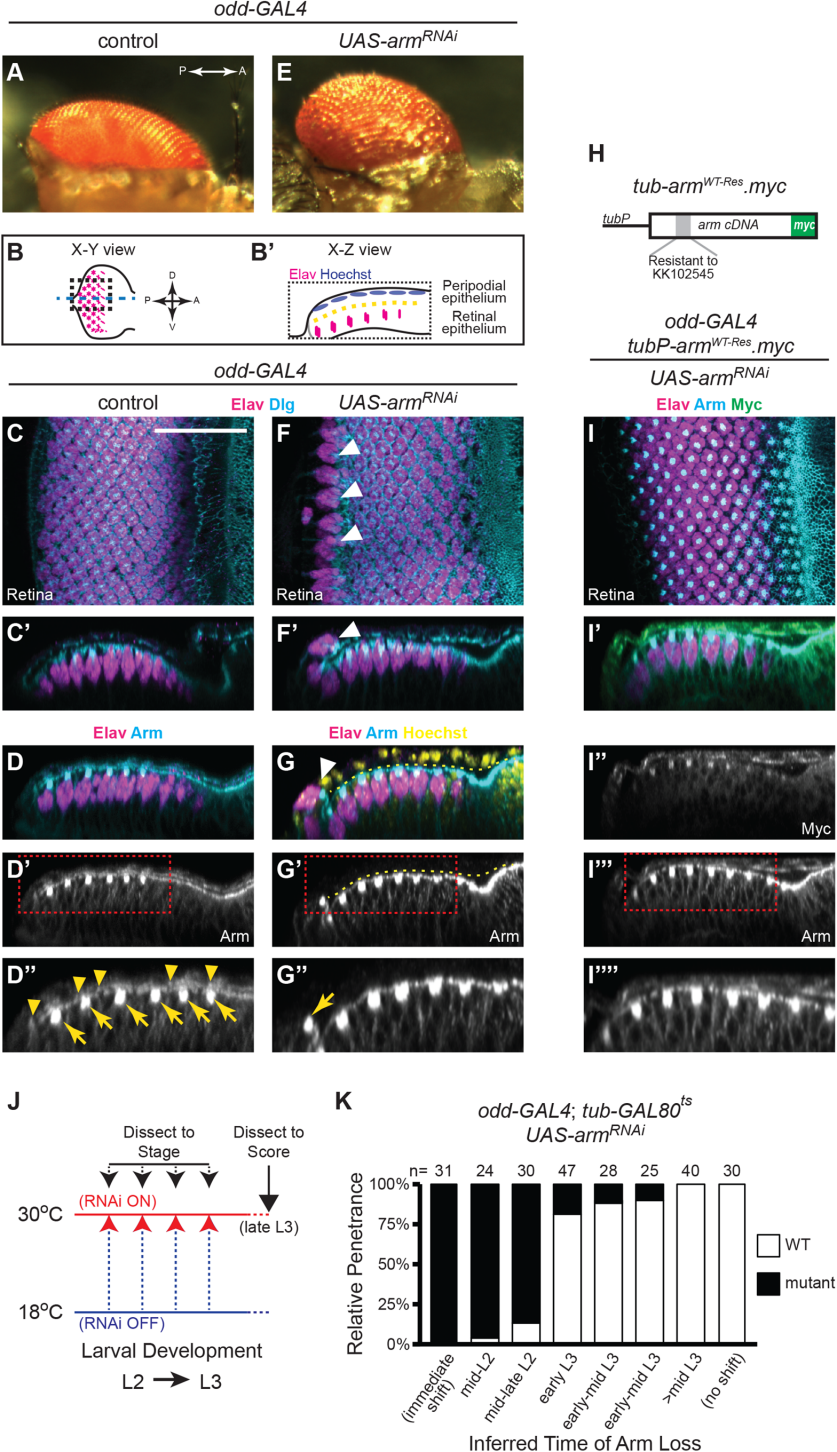
**Arm acts in the PE to preserve retinal morphology.** (A) Micrograph of adult compound eye. (B-B′) Schematics of eye discs, further described in Fig. S2. Developing photoreceptor neurons (pan-neural marker Elav, magenta; disc lumen, dashed yellow line). (C-D″) Elav expression in control L3 eye discs (co-stained as indicated). Armadillo (Arm, cyan) is enriched in the apical domains of cells in both the retina (yellow arrows) and PE (yellow arrowheads) (D-D″); vertices of ommatidial clusters in the retina are marked by bright immunoreactive puncta. (E) Micrograph of *odd-GAL4 UAS-arm^RNAi^* compound eye (*n*=25 observed). (F-G″) L3 eye discs from *odd-GAL4 UAS-arm^RNAi^* larvae, co-stained as indicated. Several rosettes of Elav-positive cells appear out of phase with the rest of the developing photoreceptor array (F; white arrowheads). Neural clusters are displaced into the PE plane of the disc (F′,G; white arrowheads). This phenotype is fully penetrant (*n*>100). Arm staining (cyan) is absent from the PE of *odd-GAL4 UAS-arm^RNAi^* discs (G-G″), but remains at the vertex of the displaced Elav-positive cell cluster (yellow arrow, G″). Hoechst counterstain (yellow) is used to aid in disc visualization. (H) Diagram of the *tubP-arm^WT-Res^.myc* transgene (see Materials and Methods). (I-I″″) *odd-GAL4 UAS-arm^RNAi^* discs that co-express the *tubP-arm^WT-Res^.myc* transgene do not exhibit ectopic Elav-positive cells in the PE (I-I′, *n*=22); transgene expression is indicated by Myc immunoreactivity (green, I′); Arm immunoreactivity (cyan) is restored to PE cells (I‴-I″″). Scale bar: 25 µm, shown in panel C, applies to images C-I″″, with the exception of the magnified panels (D″,G″,I″″). (J) Schematic of the temperature-shift paradigm used to temporally express *UAS-arm^RNAi^* in the PE (see Results). (K) Relative penetrance of the mutant phenotype relative to the inferred time of Arm depletion (see Materials and Methods).

The compound eye of *Drosophila* is composed of an array of more than 700 precisely positioned individual simple eyes, or ommatidia, and exhibits a characteristic curvature (Fig. 1A). This architecture is essential for normal fly vision (Borst, 2009). The patterning that underlies this stereotypical wild-type (wt) ommatidial arrangement is already apparent in the larval eye disc, the progenitor tissue of the compound eye (Fig. 1B). The eye disc is a flattened vesicle of apically apposed epithelial cells; one layer comprises the columnar retinal epithelium and the other, the squamous PE (Fig. 1B′). Starting in the third and final larval stage (L3), rows of neuronal clusters are progressively formed in the retina to generate a two-dimensional crystalline lattice. These precisely arrayed developing ommatidia can be visualized using the pan-neural nuclear marker Elav (Fig. 1C; *n*>100, where *n* is total number of eye discs analyzed). As seen in orthogonal views (X-Z optical sections), neurogenesis is restricted to the retinal epithelial layer of the disc (Fig. 1C′-D), whereas Arm is expressed in both layers of the disc (Fig. 1D-D″).

As part of a large RNAi screen for genes affecting eye development, we were led to investigate the function of the essential gene *arm*. Whereas globally reducing *arm* expression in the eye disc severely compromised disc development (not shown), RNAi-mediated silencing of *arm* specifically in the PE (*odd-GAL4 UAS-arm^RNAi^*=*odd*>*arm^RNAi^*) resulted in abnormal eyes (Fig. 1E, compare to wt eye in A; *n*=25, 100% penetrance). To identify the developmental origin of this phenotype we examined late-L3 *odd*>*arm^RNAi^* eye discs (Fig. 1F-G″). In contrast to wt controls (Fig. 1C), we observed clusters of Elav-positive cells at the disc posterior that appeared to be out of phase with the rest of the developing photoreceptor array (Fig. 1F, white arrowheads). When viewing X-Z optical sections it was apparent that neurons were present on the PE side of the disc (Fig. 1F′, white arrowhead; compare to C′). That the developmental abnormalities in the larval eye disc underlie the dysmorphic fly eye phenotype (Fig. 1E) is demonstrated by conditional gene-silencing experiments, described below.

To confirm that this phenotype reflected the loss of Arm, we first showed that an independent RNAi reagent, JF01252, induced the same phenotype when expressed in PE cells (Fig. S1A-B‴), albeit with 88% penetrance (*n*=56). Given the 100% penetrance achieved with *UAS-arm^RNAi^* (KK102545, used above), this reagent was used henceforth. Second, we confirmed the loss of Arm immunoreactivity in *odd*>*arm^RNAi^* discs (compare control in Fig. 1D-D″ to *odd*>*arm^RNAi^* in Fig. 1G-G″). Arm-rich AJs are present in wt PE (yellow arrowheads, Fig. 1D″) and retinal layer of the disc (yellow arrows, Fig. 1D″), but are specifically absent in the PE of *odd*>*arm^RNAi^* discs (Fig. 1G′-G″). Third, we used a constitutively expressed RNAi^KK102545^-resistant *arm* transgene, *tub-arm^WT-Res^.myc* (Fig. 1H) to rescue the mutant phenotype of *odd>arm^RNAi^* discs (Fig. 1I-I″″; *n*=22, 100% rescue). Taken together, these data establish a role for Arm in the PE of the eye disc, where it functions to preserve overall eye disc morphology.

To determine the developmental stage at which Arm function is required, we conditionally silenced *arm* in the larval eye disc. These experiments used temperature shifts and constitutively expressed temperature-sensitive GAL80 (*tub-GAL80^ts^* transgene) to modulate the activity of GAL4, and thus *UAS-arm^RNAi^* expression. Temperature shifts from 18°C (active GAL80, inactive GAL4, no RNAi) to 30°C (inactive GAL80, active GAL4, RNAi present) were used to induce the loss of Arm protein at different stages of larval development, starting in L2 and through late L3 (Fig. 1J). Disc developmental stage at the time of Arm loss was inferred by staging discs at the time of dissection, and correcting for the rate of loss of Arm protein at AJs, and for the rate of development of larvae of this genotype (see Materials and Methods).

As expected, neurons were not observed in the PE of *odd*>*arm^RNAi^* (*tub-GAL80^ts^*) negative control samples kept at 18°C throughout development (*n*=30), whereas positive controls shifted to 30°C immediately after egg collection exhibited the phenotype with 100% penetrance (*n*=31) (Fig. 1K). Loss of Arm in discs prior to the start of neurogenesis (in mid or mid-to-late L2) displayed the mutant phenotype with high penetrance (96%, *n*=24; 87%, *n*=30) (Fig. 1K). On the contrary, Arm loss in early to mid-early L3 discs (<7 rows of developing ommatidia) induced the mutant phenotype with much lower penetrance (19% in very early L3, *n*=47; 10-12% in early to mid-early L3, *n*=28 and *n*=25, respectively) (Fig. 1K). After larvae reached the wandering stage (>mid-L3), shifting them to 30°C for the remainder of L3 did not induce abnormalities in the larval eye disc (*n*=40) or the fly eye (not shown). These results point to a requirement for Arm function prior to that start of neurogenesis in early L3, and indicate that the eye disc abnormalities underlie the dysmorphic fly eye phenotype. Thus, Arm is required in the PE to promote normal retinal epithelium and adult eye morphology.

### Neurons in the PE plane of *odd>arm^RNAi^* discs result from a shift of the retinal epithelium

We have recently reported two distinct mechanisms that can result in the detection of neurons on the PE side of the eye disc (Neal et al., 2020; 2022). We thus performed a series of experiments, including a developmental analysis and lineage tracing of PE cells in *odd>arm^RNAi^* discs, to distinguish between PE cell fate change and retinal displacement (RDis) phenotypes.

**Fig. 2.**
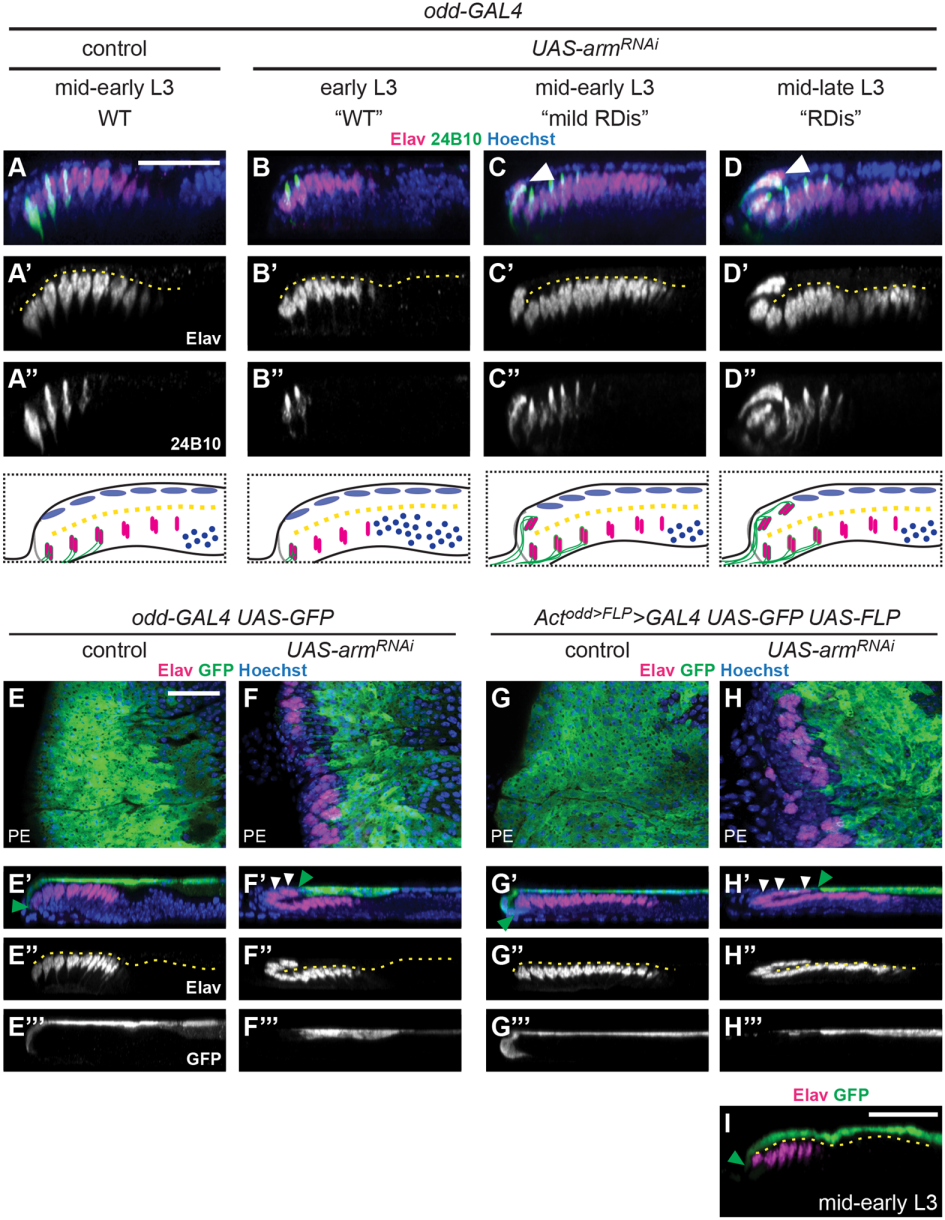
**Neurons are progressively mislocalized from the retina into the PE plane in *odd>arm^RNAi^* discs.** (A-D″) Chaoptin expression (24B10 antigen, green), marks more mature Elav-positive (magenta) ommatidial clusters in *odd-GAL4* control discs (A-A″), as in *odd-GAL4 UAS-arm^RNAi^* discs, early in retinal development (B-B″). As retinogenesis proceeds in *odd-GAL4 UAS-arm^RNAi^* discs, the posterior-most Elav+24B10-positive cells clusters become progressively mislocalized to the PE plane of the disc (C-D″). Developmental stages as indicated; phenotypes are schematized below. (E-F‴) *odd-GAL4* control (E-E‴) and *UAS-arm^RNAi^* discs (F-F‴), co-expressing *UAS-GFP* (green), to mark the *odd* expression domain; boundary indicated by green arrowheads (E′,F′). GFP immunoreactivity labels the PE plane of the control discs, and does not co-stain Elav-positive cells (magenta) in the retinal plane (E-E‴). GFP expression is absent from the posterior of *odd>arm^RNAi^* discs, where ectopic Elav cells (white arrowheads) are present (F-F‴). (G-I) *UAS-GFP* marks the expression domain (green; G-G′, H-H′) of constitutive *odd-GAL4* expression (see Results). In control discs (G-G‴), GFP expression in the PE is more uniform, but follows the same pattern as for *odd-GAL4* alone (compare with E-E′, E‴). *Act^odd>FLP^>GAL4 UAS-arm^RNAi^* discs exhibited a slightly more severe RDis phenotype, with more Elav-positive cell clusters present in the PE plane of the discs (H-H′, white arrowheads). These clusters did not co-stain for GFP (H-H‴). Developmentally earlier *Act^odd>FLP^>GAL4 UAS-arm^RNAi^* discs are wt in appearance (I). Green arrowheads mark the boundary of GFP expression (G′,H′,I). Scale bars: 25 µm, shown in panels A, E and I apply to panels A-D‴, E-H‴ and I, respectively. Hoechst counterstain (blue) is used throughout to aid in disc visualization; dashed yellow lines indicate disc lumens.

In developing wt retinae, neurogenesis initiates at the disc posterior and propagates anteriorly by progressively adding rows of newly formed neuronal clusters. As such, more anterior (younger) neurons express Elav alone, while Elav-positive ommatidial clusters at the posterior are developmentally older and co-express the photoreceptor-specific 24B10 antigen (Fig. 2A-A″). Interestingly, we observed progression of the mutant phenotype as retinal neurogenesis proceeded in *odd>arm^RNAi^* discs (Fig. 2B-D″). The mutant phenotype was never observed in early L3 *odd>arm^RNAi^* discs, typically having fewer than eight rows of Elav-positive ommatidial clusters (Fig. 2B-B″, Fig. S1C-F). With respect to Elav staining, these discs were indistinguishable from wt controls (discs expressing *odd-GAL4* alone); Elav expression was restricted to the retinal plane and, in discs with 5-7 rows of Elav-positive clusters, 24B10 expression marked only the most posterior 1-4 rows of clusters (Fig. 2B-B″). In more developed *odd>arm^RNAi^* L3 discs, a distinctive curling of the retinal array was sometimes observed at the disc posterior (Fig. 2C-C″, white arrowhead; Fig. S2C-D, G-H), and by mid-late L3, the mutant phenotype was fully penetrant (Fig. 2D-D″, white arrowhead; Fig. S2C-D, I-J). In the latter discs, all the mislocalized neurons co-expressed 24B10 and Elav (Fig. 2D-D″). Based on these observations, we conclude that the neurons in the PE domain of *odd>arm^RNAi^* discs are likely to be retinal neurons.

We next employed cell labeling strategies to define cells of PE origin in *odd>arm^RNAi^* discs. We first examined contemporary *odd-GAL4 UAS-GFP* expression in wt and *arm^RNAi^* discs (Fig. 2E-F‴). In wt L3 discs the *odd* expression domain spans most of the PE; in particular, a clear GFP expression boundary is observed between the PE and retinal domains of the disc epithelium (Fig. 2E-E‴, green arrowhead). Interestingly, the boundary between GFP-expressing and non-expressing domains of the eye disc was shifted onto the PE side of the disc in *odd*>*arm^RNAi^* larvae (Fig. 2F-F‴, green arrowhead). Notably, GFP expression was absent from Elav-positive cells, including those found in the PE domain (Fig. 2F-F‴,white arrowheads). Furthermore, we note that the displaced neuronal clusters also continue to express Arm (Fig. 1G″, yellow arrow).

We repeated this experiment using a compound driver that converts the developmentally regulated *odd-GAL4* driver into a constitutive one (*Act^odd>FLP^>GAL4*), enabling us to constitutively label cells of PE origin. This driver relies on *odd-GAL4*-driven *UAS-FLP* expression to excise the interruption cassette (IC) from an *Act5C>IC>GAL4* transgene. Thus, UAS transgenes can be expressed constitutively in the PE under control of the *Act5C-GAL4*, mitigating confounds from any differential regulation of the *odd* locus in response to *arm^RNAi^* expression. In control discs with *UAS-GFP* but no RNAi, the wt GFP expression pattern was observed (Fig. 2G-G‴, green arrowhead, compare to E-E‴). In experimental samples with *UAS-GFP* and *UAS-arm^RNAi^*, the boundary marked by GFP was again shifted onto the PE side of the disc (Fig. 2H-H‴, green arrowhead), and GFP expression was notably absent from all of the Elav-positive cells in the PE (white arrowheads). In short, *Act^odd>FLP^>GAL4 UAS-arm^RNAi^ UAS-GFP* discs were indistinguishable from *odd-Gal4 UAS-arm^RNAi^ UAS GFP* discs. Furthermore, younger discs of this genotype were wt in appearance, having their GFP expression boundary at the posterior margin of the disc (Fig. 2I, green arrowhead), and lacking displaced neurons.

Collectively, these observations suggest that neurons in the PE domain of *odd*>*arm^RNAi^* discs do not arise *de novo* from PE cells. Rather, the phenotype reflects a shift of the eye disc epithelium such that the PE-retinal boundary is no longer at the disc posterior margin. Altogether, these finding demonstrate that the observed phenotype is RDis (Fig. S2A-C″).

### Disruption of AJs in the PE leads to RDis

**Fig. 3.**
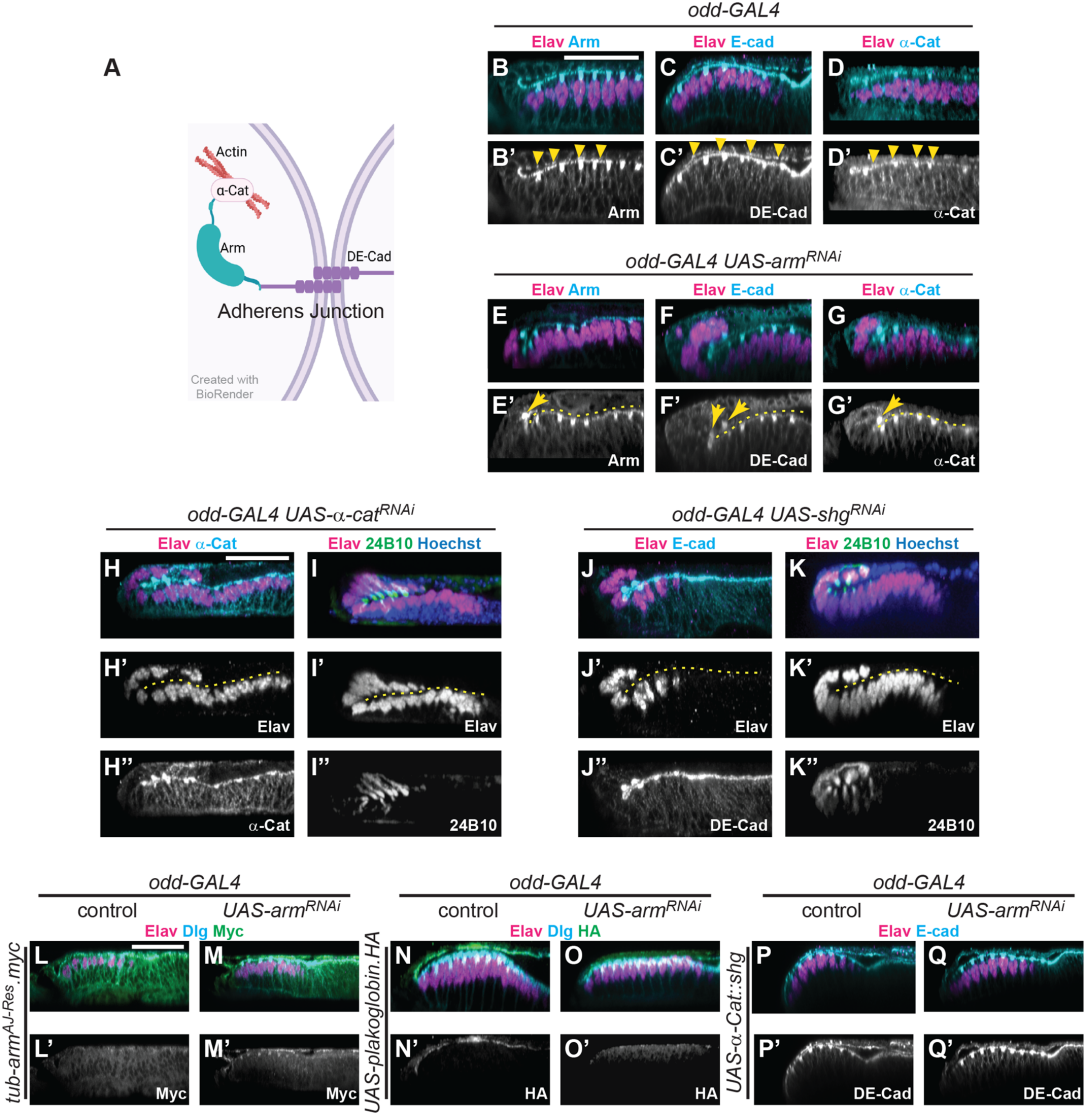
**AJs, but not specifically Arm, are necessary in the PE to prevent retinal displacement.** (A) Schematic of an AJ, created with BioRender. (B-D′) Expression of AJ components in wild-type (*odd-GAL4*) eye discs. Arm (B-B′), DE-Cad (C-C′) and α-Cat (D-D′) (cyan, in respective panels) are present and apically enriched in both layers of the eye disc (yellow arrowheads indicate PE expression; Elav, magenta). (E-G′) Apical enrichment of AJ components in the PE is lost in *odd-GAL4 UAS-arm^RNAi^* discs. While Arm immunoreactivity is strongly reduced in the PE (E-E′), DE-Cad (F-F′) and α-Cat (G-G′) immunoreactivity appears more disorganized. All AJ components exhibit punctate staining at the vertices of displaced Elav-positive ommatidial clusters (yellow arrows). (H-I″) *odd-GAL4 UAS-α-cat^RNAi^* discs exhibit decreased α-Cat immunoreactivity in the PE (cyan, H-H″) and displaced Elav-positive cell clusters (magenta) co-stain for the 24B10 antigen (green, I-I″; *n*=15, 100% penetrant). Similarly, *odd-GAL4 UAS-shg^RNAi^* discs exhibit decreased DE-Cad immunoreactivity in the PE (cyan, J-J″) and displaced Elav-positive cell clusters (magenta) co-stain for the 24B10 antigen (green, K-K″; *n*=22, 100% penetrant). (L-M′) Constitutive expression of transcriptionally compromised Arm (*tub-arm^AJ-Res^.myc* transgene, see Materials and Methods) does not affect wild-type eye disc development (A-A′; *n*=15) but fully suppresses RDis in *odd-GAL4 UAS-arm^RNAi^* discs (M-M′; *n*=20). Myc immunoreactivity (green) is apically enriched in both cell layers of the disc. (N-O′) Expression of *UAS-plakoglobin-HA* in the PE, driven by *odd-GAL4*, does not affect wt disc development; Elav-positive cells (magenta) (N-N′; *n*=15). In discs expressing *arm^RNAi^*, the RDis phenotype is fully rescued by Plakoglobin expression (O-O′; *n*=25), marked by HA immunoreactivity (green), restricted to the PE cell layer (N-O′). (P-Q′) Expression of an α-Cat::DE-Cad fusion protein (*UAS-::α-Cat::shg* transgene) did not affect wild-type disc development (P-P′; *n*=15), but fully rescued the RDis phenotype in *odd-GAL4 UAS-arm^RNAi^* discs, as indicated by Elav expression (magenta) (Q-Q′; *n*=30). DE-Cad immunoreactivity (cyan) was used to detect expression of the transgene, in addition to endogenous DE-Cad. Scale bars: 25 µm, shown in panels B, H, and L, apply to panels B-G′, H-K″, and L-Q′, respectively. Hoechst counterstain (blue) is used, as indicated, to aid in disc visualization; dashed yellow lines indicate disc lumens.

One essential role of Arm in epithelia is at AJs, where Arm bridges the cell–cell adhesion factor DE-Cad and the intracellular cytoskeletal connector α-Cat (Fig. 3A). Thus, Arm serves as a critical link in a ‘Cadherin–Catenin chain’ that connects the cytoskeletons of neighboring cells, throughout an epithelium (Harris and Tepass, 2010). Interestingly*,* the localization of constituent proteins to AJs is highly interdependent. Silencing of *α-Cat* (*α-Cat^RNAi^*) in wing discs was previously shown to result in the mislocalization of Arm and DE-Cad, whereas α-Cat was mislocalized when DE-Cad was knocked down (*shg^RNAi^*) in this tissue (Yang et al., 2015). Given the importance of junctions in epithelia, we sought to establish whether a disruption of AJs, as opposed to the specific absence of Arm, caused the RDis phenotype.

First, we examined the effects of Arm loss from PE AJs on the localization of other AJ components in the eye disc. In wt discs, Arm, DE-Cad and α-Cat are apically enriched in all cells, where punctate staining is indicative of AJs (Fig. 3B-D′, yellow arrowheads). In contrast, *odd*>*arm^RNAi^* discs (Fig. 3E-G′) not only exhibited the expected loss of Arm from the PE (compare B′ with E′), but also the mislocalization of both DE-Cad and α-Cat (compare C′ with F′, and D′ with G′, respectively). In all cases, AJ component expression was maintained at the apices of mislocalized Elav-positive cell clusters (yellow arrows, Fig. 3E′, F′, G′). Hence, AJs are grossly perturbed in the PE of *odd*>*arm^RNAi^* discs.

To investigate whether RDis resulted from the specific loss of Arm or a more general role for AJs, we asked whether loss of other AJ components in the PE could phenocopy loss of Arm. Using *odd-GAL4*, we induced the loss of either α-Cat (*α-Cat^RNAi^*, *n*=15, Fig. 3H-I″) or DE-Cad (*shg^RNAi^*, *n*=22, Fig. 3J-K″) from the PE. Expression of *α-Cat^RNAi^* decreased α-Cat immunoreactivity (compare H″ with D′) and resulted in the RDis phenotype with 100% penetrance; all ommatidial clusters on the presumptive PE side of the disc expressed the 24B10 antigen (Fig. 3I-I″). Expression of *shg^RNAi^* similarly induced a fully penetrant RDis phenotype (Fig. 3J-K″). In these discs, DE-Cad immunoreactivity was lost from the PE (compare J″ with C′), and mislocalized Elav-positive cells were 24B10-positive (Fig. 3K-K″).

These data suggest that the disruption of AJs, rather than the loss of Arm *per se*, causes the RDis phenotype. Hence, we performed additional experiments to assess whether the junctional function of Arm was sufficient to ensure normal disc morphology.

### AJs, but not Arm, are required in the PE for normal disc morphology

Arm is known to play many critical roles during development, including as a transcriptional effector of Wnt signaling (Bejsovec, 2018; Clevers and van de Wetering, 1997). Hence, to demonstrate the centrality of its role at the AJ in the etiology of the RDis phenotype, we sought to reestablish AJs in *odd*>*arm^RNAi^* discs using reagents that had little or no transcriptional function. Three such reagents have been developed for use in *Drosophila*: (1) a transgene that ubiquitously expresses a transcriptionally-compromised form of Arm (*tub-arm^AJ^.myc*); (2) a transgene that expresses Plakoglobin/γ-Catenin under UAS control (*UAS-Plakoglobin.HA*), and (3) a transgene that expresses an α-Cat::DE-Cad fusion protein under UAS control (*UAS-α-Cat::shg*). Arm^AJ^ is C-terminally truncated and has a single point mutation (D172A) in the Arm-repeat domain; together, these mutations abrogate its interaction with a multitude of transcriptional co-activators (Valenta et al., 2011). Plakoglobin is an atypical member of the mammalian β-Catenin family that can take the place of Arm at AJs, but not in the nucleus (White et al., 1998). Lastly, α-Cat::DE-Cad has been shown to bypass the requirement for β-Catenin altogether in functionally restoring AJs (Pacquelet and Rorth, 2005; Sarpal et al., 2012).

Ubiquitous expression of Arm^AJ^.myc, which we modified to be RNAi resistant (*tub-arm^AJ-Res^.myc* transgene), had no effect on wt eye disc development (Fig. 3L-L′, *n*=15), but was able to completely rescue the RDis phenotype in discs expressing *odd*>*arm^RNAi^* (Fig. 3M-M′, *n*=20). Similarly, expression of Plakoglobin in the PE using *odd-GAL4* did not affect control disc development (Fig. 3N-N′, *n*=15), but completely suppressed the RDis phenotype in *odd*>*arm^RNAi^* discs (Fig. 3O-O′, *n*=25). Plakoglobin expression, marked by HA immunoreactivity, was observed only in the PE domain (Fig. 3N′,O′). Finally, expression of the α-Cat::DE-Cad fusion protein in the PE did not affect control disc development (Fig. 3P-P′, *n*=15), but was also able to completely suppress the *odd*>*arm^RNAi^* RDis phenotype (Fig. 3Q-Q′, *n*=30).

In summary, we were able to rescue disc morphology in *odd*>*arm^RNAi^* discs by restoring AJs independently of Arm. These findings support a model whereby RDis results from the disruption of AJs, and not simply loss of Arm.

### AJs in the PE regulate Yki signaling to prevent RDis

Beyond their structural role, AJs are also associated with the regulation of Yki, the key downstream effector of the Hippo signaling cascade (Oh and Irvine, 2010). The Hippo cascade kinase Wts directly phosphorylates Yki, resulting in its sequestration in the cytoplasm by 14-3-3 proteins, and thus impeding the transcription of Yki-dependent target genes (Misra and Irvine, 2018; Pfleger, 2017). However, this function of Wts is antagonized by the AJ-associated protein Jub, which can sequester Wts at AJs and prevent it from phosphorylating Yki (Alegot et al., 2019; Rauskolb and Irvine, 2019; Sarpal et al., 2019). Jub-dependent negative regulation of Wts fails when AJs are disrupted.

We have recently shown that Yki is central to suppressing RDis (Neal et al., 2022). In fact, inducing the loss of Yki from the late-L2 stage PE results in a severe RDis phenotype. Since AJs play a positive role in the regulation of Yki activity, we reasoned that the requirement for AJs in maintaining proper disc morphology might reflect a requirement for Yki function. To test this hypothesis, we investigated two predictions: (1) loss of Jub from the PE (like loss of Arm, DE-Cad, α-Cat, or Yki) should result in RDis; and (2) overexpression of Yki may overcome negative regulation by Wts and rescue the RDis phenotype of *odd>arm^RNAi^* discs.

**Fig. 4.**
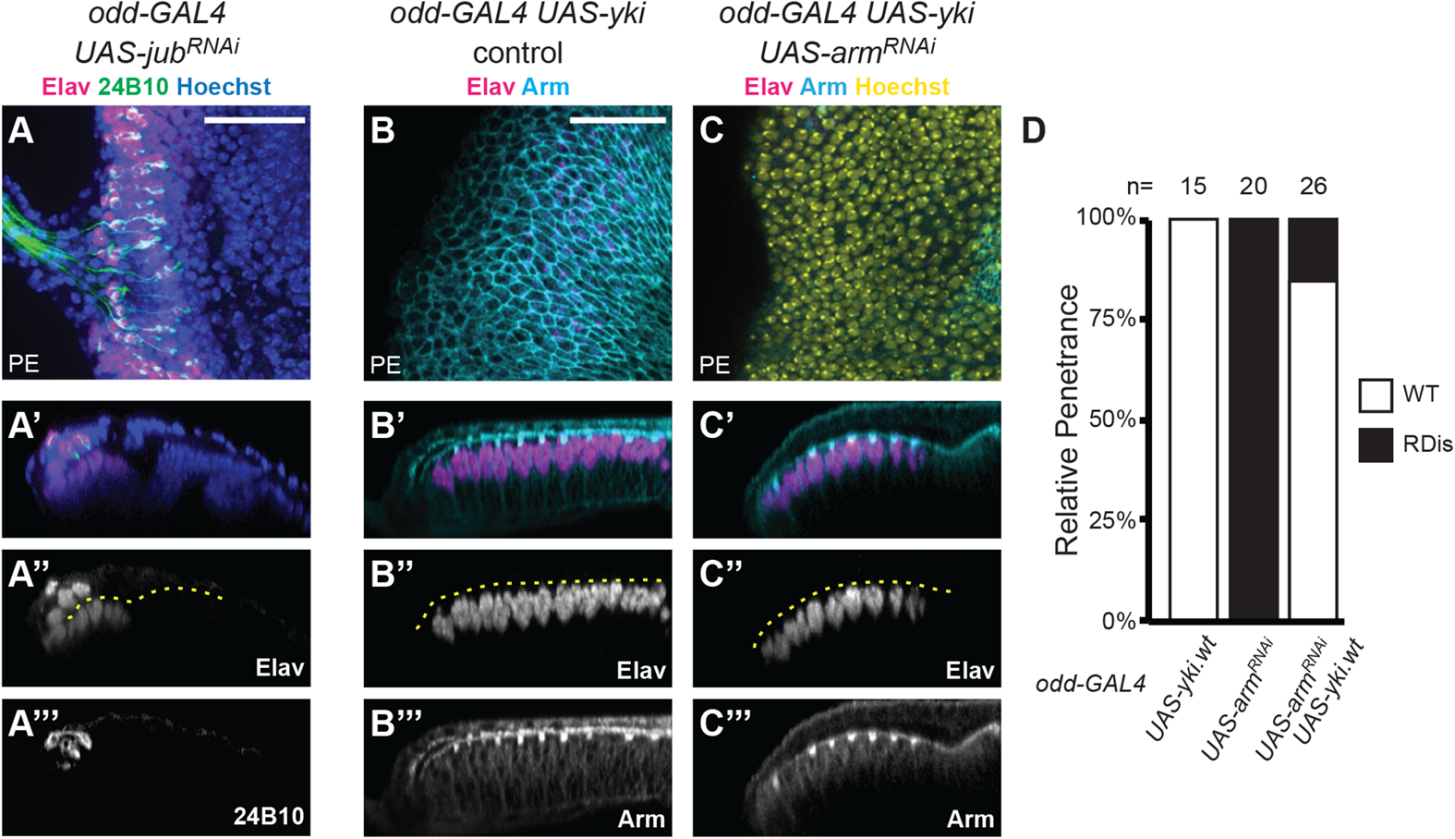
**AJ-Jub-Yki signaling in the PE prevents retinal displacement.** (A-A‴) *odd-GAL4 UAS-jub^RNAi^* discs exhibit the RDis phenotype, as illustrated by the presence of Elav (magenta)+24B10 (green) immunoreactive cell clusters in the PE plane of the eye disc (*n*=20). (B-C‴) Overexpression of *UAS-yki* in the PE of control discs using *odd-GAL4* did not grossly affect disc morphology (B-B‴; *n*=15). The retinal marker Elav (magenta) was not detected in the PE, where Arm expression (cyan) marks apical cell–cell junctions (B-B″). In discs expressing *UAS-arm^RNAi^* in the PE, RDis was suppressed by *UAS-yki* expression; apically-enriched Arm immunoreactivity was not restored in these discs (C-C‴). (D) Relative penetrance of the RDis phenotype in discs of the indicated genotypes (*n* as indicated). Scale bars: 25 μm, in panels A and B apply to panels A-A‴ and B-C‴, respectively. Hoechst counterstain (yellow) is used, as indicated, to aid in disc visualization; dashed yellow lines indicate disc lumens.

To address our first hypothesis, we expressed *UAS*-*jub^RNAi^* in the PE using *odd*-*GAL4.* To mitigate early larval lethality, we prevented *jub^RNAi^* expression prior to the L2 stage using GAL80^ts^. Under these conditions, all *odd*>*jub^RNAi^* discs (*n*=20) showed the RDis phenotype, with 24B10-positive neuronal clusters in the PE plane (Fig. 4A-A‴). Thus, loss of Jub, just like loss of AJs (Fig. 3), results in RDis.

To extend this connection to Yki, and address our second hypothesis, we tested whether increasing the level of Yki could rescue the 100% penetrant RDis phenotype of *odd>arm^RNAi^* discs. Driving a *UAS-yki.wt* transgene (Huang et al., 2005) with *odd-GAL4* in the absence of *arm^RNAi^*, did not induce eye disc abnormalities (Fig. 4B-B‴, *n*=15). Yet, in *odd>arm^RNAi^* discs, Yki expression effectively suppressed RDis (Fig. 4C-C‴), and did so without restoring Arm expression in the PE (Fig. 4C). Only 15% of *odd>arm^RNAi^ UAS-Yki* discs displayed the RDis phenotype; 22/26 discs were indistinguishable from wt controls (Fig. 4D).

Altogether, these findings strongly suggest that AJs, via Jub, preserve disc and retinal morphology during larval development by promoting an appropriate level of Yki activity in PE cells. Thus, with respect to RDis, the critical role of the AJ in the PE is that of a signaling hub rather than of a cell–cell adhesion complex.

### RDis is not caused by a decrease in the number of PE cells

**Fig. 5.**
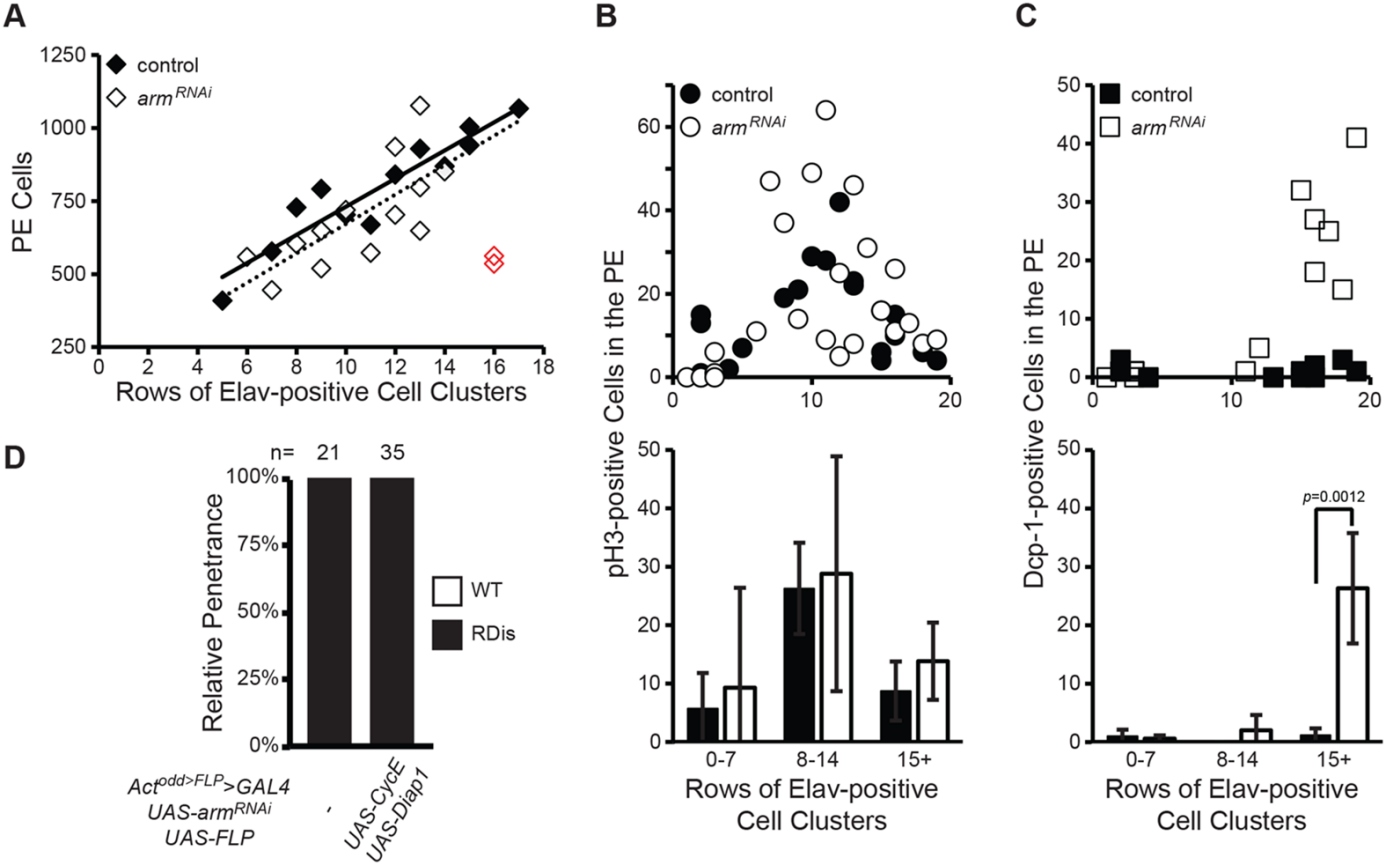
**PE cell number, mitosis and apoptosis are unaffected as RDis becomes penetrant.** (A-C) Quantification of PE cell number (A), mitotic cells (B), and apoptotic cells (C) in *odd-GAL4* control (filled symbols) and *UAS-arm^RNAi^* (open symbols) discs, as a function of the progression of retinal development in L3 (open red markers – see Results). Cell number was counted in discs stained for the basolateral membrane marker Dlg (A). Mitotic cells were scored on the basis of phospho-histone H3 (pH3) immunoreactivity (B). Apoptotic cells were scored on the basis of Dcp-1 immunoreactivity (C). Student's *t*-tests were performed on binned data (lower panels in B and C; see Fig. S1). (D) Co-expression of pro-proliferative (*UAS-CycE*) and anti-apoptotic (*UAS-Diap1*) factors in *odd-GAL4 UAS-arm^RNAi^* discs does not suppress RDis.

Yki is a well know regulator of diverse cellular processes, most prominently of proliferation and cell death (Oh and Irvine, 2010; Pan, 2010). Given the identification of Yki as a critical effector downstream of AJs and the obvious decrease in PE surface area in affected discs, we sought to assess whether a decrease in PE cell number, induced by either decreased proliferation or increased cell death, underlies the RDis phenotype. Hence, we quantified cell numbers in the PE (Fig. 5A), as well as proliferation (Fig. 5B) and apoptotic cell death (Fig. 5C). Since the eye disc continues to grow during the L3 stage, while the developing retina expands by one row of neuronal clusters at a time, we used the number of rows of Elav-positive neuronal clusters as a proxy for developmental stage (Campos-Ortega, 1980).

In L3 discs having between 5 and 14 rows of Elav-positive cell clusters, representing both pre-RDis and RDis discs in the case of *odd*>*arm^RNAi^* larvae (Fig. S1C-D), we did not observe gross differences in PE cell numbers (Fig. 5A). Based on the slopes of linear trend lines, approximately 50 cells were added to the PE per row of Elav-positive cell clusters added to the retina, in discs of both genotypes. Consistent with the observed conservation in cell numbers, we did not observe significant changes in proliferation or cell death. The number of pH3-positive cells detected in the PE, while typically low and variable, was not significantly different between the genotypes in pre-RDis discs (0-7 rows), nor in older discs in which the RDis phenotype first becomes penetrant in the *arm^RNAi^* condition (8-14 rows) (Fig. 5B). Similarly, we did not detect a change in rates of cell death. Apoptotic cells in the PE were rare in discs having 0-7 rows or 8-14 rows of Elav-positive cell clusters (Fig. 5C). Thus, relative to control discs, in both pre-RDis (0-7 rows) and RDis (8-14 rows) *odd*>*arm^RNAi^* discs, there are no significant differences in the number of cells, mitotic cells, or apoptotic cells in the PE.

In two older *odd*>*arm^RNAi^* discs (15+ rows), we did observe a precipitous drop in PE cells number (Fig. 5A, red markers). This paralleled an observed increase in the number of apoptotic cells in discs of this genotype having 15+ rows of Elav-positive cell clusters (Fig. 5C; *P*=0.0012); no significant change in the number of mitotic cells was observed in these discs (Fig. 5B). Because *odd*>*arm^RNAi^* discs at this stage are >30 h into neurogenesis and have expressed the RDis phenotype for up to 16 h (Campos-Ortega, 1980), we consider cell death at this stage to be a secondary consequence of the RDis phenotype, or its underlying mechanisms.

In agreement with these findings, RDis in *odd>arm^RNAi^* discs was not rescued by co-overexpression of *CyclinE* (*CycE*) and *Drosophila* Inhibitor of Apoptosis (*Diap1*), two Yki target genes previously shown to counteract the effect of compromised Hpo-Yki signaling on proliferation and cell death in the eye disc (Neal et al., 2020; Kenyon et al., 2003; Zhang et al., 2011). When *CycE* and *Diap1* were constitutively co-expressed with *arm^RNAi^* in the PE (via *Act^odd>FLP^>GAL4*), the RDis phenotype remained fully penetrant (*n*=35; Fig. 5D).

Taken together, these data show that emergence of the RDis phenotype is not accompanied by changes in the rates of proliferation or cell death in the PE, nor is it due to a decrease in PE cell number. We thus considered additional mechanisms which could affect epithelial morphology.

### Involvement of the extracellular matrix and cellular tension in disc morphology and RDis

**Fig. 6.**
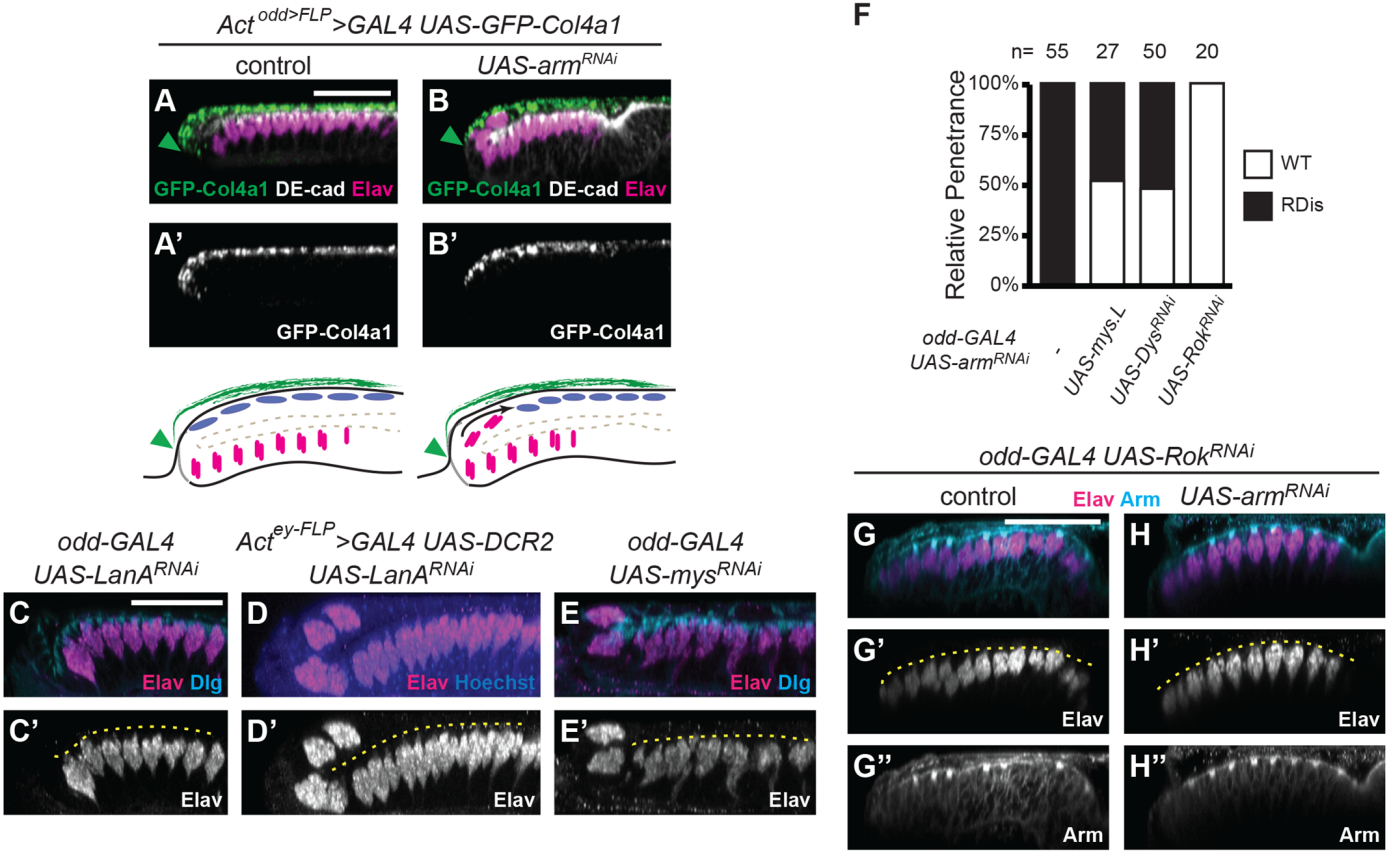
**PE-ECM attachment and PE cell tension are perturbed in RDis discs.** (A-B′) Constitutive expression (*Act^odd>FLP^-GAL4*) of the M-TRAIL reagent (*UAS-Col4a1-GFP*) in PE cells of control (A-A′) and *UAS-arm^RNAi^* (B-B′) discs marks the ECM of the PE cell layer (green); DE-Cad (white) and Elav (magenta) staining is also shown. Diagrams to the right illustrate how the disc epithelium slips beneath the PE ECM in RDis. (C-C′) PE-specific knockdown of LanA (*odd>LanA^RNAi^*) did not affect disc morphology [*n*=16/16 wt discs; Elav-positive cell clusters (magenta); Dlg staining (cyan)]. (D-D′) Disc-wide knockdown of LanA (*Act^ey-FLP^-GAL4>LanA^RNAi^*) yielded the RDis phenotype with low penetrance [*n*=14/54 RDis discs; Elav-positive cell clusters (magenta); Hoechst counterstain (blue)]. (E-E′) Knockdown of the βPS-integrin in the PE (*odd>mys^RNAi^*) results in displaced Elav-positive cell clusters (magenta); Dlg staining (cyan). (G) Quantification of RDis penetrance in discs of the indicated genotypes; *n* as shown. (G-H″) *odd-GAL4* control (G-G″) and *UAS-arm^RNAi^* discs (H-H″) co-expressing *UAS-Rok^RNAi^*; the RDis phenotype was completely suppressed in the latter (*n*=20). Discs are stained for Elav (magenta) and Arm (cyan). Scale bars: 25 μm, in panels A, C and G apply to panels A-B′, C-E′ and G-H″, respectively.

The extracellular matrix (ECM) is an essential contributor to epithelial morphology (Pastor-Pareja and Xu, 2011; Bonche et al., 2022). For instance, ECM components Collagen IV and Perlecan in the PE of the wing disc have very recently been demonstrated to affect disc morphogenesis (Bonche et al., 2022). Thus, to better understand how an apparent shift in the disc epithelium related to the disc ECM, we used the ‘M-TRAIL’ reagent *UAS-Col4a1-GFP*, which encodes a secreted Collagen IV-GFP fusion protein (Chen et al., 2017). This allowed us to assess the behavior of the epithelium vis-à-vis the underlying ECM. Using the *Act^odd>FLP^-GAL4* driver, we were able to specifically drive expression of Collagen IV-GFP in PE cells. Studies have shown that at least some ECM components originate from distant tissues, e.g. fat body, as well as being produced locally (Pastor-Pareja and Xu, 2011). In the case of the eye disc, Collagen IV-GFP expressed in, and secreted by, PE cells was integrated efficiently into the local ECM (Fig. 6A-A′). This resulted in the marking of the ECM underlying the PE portion of the eye disc epithelium but not the ECM on the retinal side. This expression domain mirrors the epithelial domain marked in PE lineage-tracing experiments (Fig. 2G′), and abuts the unmarked neuronal field of the retina (Fig. 6A, green arrowhead).

Interestingly, in discs expressing *odd*>*arm^RNAi^* and having the RDis phenotype (Fig. 6B-B′), the Collagen IV-GFP-marked ECM and the PE do not remain in register. Instead, the labelled ECM underlies the basal side of the displaced portion of the retina (Fig. 6B). We believe this reflects the incorporation of Collagen IV-GFP into the ECM during the developmental period prior to RDis, and the subsequent sliding of the retinal epithelium around the margin and anteriorly over the GFP-marked ECM, as the RDis phenotype develops (see diagrams to the right of Fig. 6A′,B′). This experiment not only confirmed the tissue-level mechanism leading to RDis, involving sliding of the eye disc epithelium, but also suggests that the attachment of the epithelial cells to their underlying ECM might be compromised or altered in RDis discs.

To investigate whether compromised ECM-epithelium contacts play a causal role in RDis, we used an RNAi-mediated knockdown approach to assess the contribution of ECM-associated factors to eye disc morphology. Epithelium-ECM attachment is primarily mediated by two transmembrane protein complexes, integrin complexes (ICs) and dystrophin glycoprotein complexes (DGCs). These complexes can both bind laminin in the ECM, and contribute to epithelial morphology, in part, by resisting certain intracellular and extrinsic forces. Whereas ECM receptors typically function in a cell autonomous manner, ECM components are produced and secreted by multiple tissues, as mentioned above. Hence, somewhat unsurprisingly, PE-restricted knockdown of the laminin α-chain (*odd-GAL4*>*LanA^RNAi^*) did not yield a phenotype (Fig. 6C-C′, 16/16 wt discs). However, a broader approach, involving the constitutive knockdown of *LanA* throughout the eye disc (*Act^ey-FLP^*>GAL4>*LanA^RNAi^*), yielded the RDis phenotype with limited penetrance (14/54 RDis discs, Fig. 6D-D′). Thus, compromising the ECM surrounding the eye disc is sufficient to induce RDis.

Notably, analyses of ICs and DGCs implicated these laminin-interacting transmembrane complexes in maintaining the morphology of the eye disc at the L3 stage. In the case of ICs, PE-specific knockdown of the βPS-integrin subunit, encoded by *mys*, resulted in the RDis phenotype in *odd>mys^RNAi^* discs (Fig. 6E-E′, 5/14 discs). Knockdown of *mys* using *GMR46D04-GAL4*, a second and recently described PE-specific GAL4 driver (Neal et al., 2022), also resulted in the RDis phenotype with similar penetrance (14/51 discs, not shown). Moreover, PE-driven expression of Mys (*UAS-mys.L* transgene) substantially suppressed the fully penetrant RDis phenotype of late L3 *odd>arm^RNAi^* discs (Fig. 6F, 14/27 wt discs). In the case of DGCs, Dystroglycan (Dg) is known to associate with laminin in the ECM and to the intracellular cytoskeleton via Dystrophin (Dys), but neither Dg nor Dys is essential for *Drosophila* development (Christoforou et al., 2008). Accordingly, no phenotype was observed when Dg or Dys was knocked down in PE cells (not shown). However, depleting cells of Dys in the context of *arm^RNAi^* resulted in a strong reduction in RDis penetrance (Fig. 6F, 24/50 wt discs). This finding supports a role for DGCs in the maintenance of disc morphology and prompted us to examine the role of cell tension, in particular, in the RDis phenotype.

In imaginal discs, tension can be manipulated by altering the activity level of Rho kinase (Rok) (Rauskolb et al., 2014; 2019; Pan et al., 2018). Rok promotes increased contractility of the actin cytoskeleton via the non-muscle type 2 myosin regulatory light chain. Thus, decreasing Rok activity should decrease cellular tension. Interestingly, all discs co-expressing RNAi against *Rok* and *arm* were rescued for the RDis phenotype (Fig. 6F, *n*=20). Control discs (*odd>Rok^RNAi^*) were wt in appearance and expressed Arm in both the PE and retina (Fig. 6G-G″). Discs expressing both *arm^RNAi^* and *Rok^RNAi^* did not exhibit the RDis phenotype despite the absence of Arm staining in the PE (Fig. 6H-H″). This result suggests that increased contractile force within the PE likely contributes to the RDis phenotype in *odd*>*arm^RNAi^* discs.

We conclude from these experiments that integrin-mediated attachment of PE cells to the laminin-containing ECM serves to maintain the morphology of the eye disc at the L3 stage. Furthermore, our data suggest that AJs in the PE function to regulate these cell-ECM interactions and cell tension. However, our current study only briefly explores these functions as they relate to eye disc morphology. Indeed, we have made preliminary observations of PE cells in wt, RDis, and RDis-rescued tissue (Fig. S3A-J) and have noticed that there appear to be multiple solutions, with respect to cell shape, aspect ratio, and size, that accommodate normal retinal development. Additional research will be required to fully appreciate these distinct solutions.

## DISCUSSION

During *Drosophila* development, proper morphogenesis of the adult compound eye requires that the retinal and peripodial epithelia of the eye disc develop in concert. At the time of neurogenesis, the two epithelia are juxtaposed along their apical surfaces but separated by a thin lumen, whereas their basal surfaces are outwardly oriented and are surrounded by basement membrane. Over the past 2 decades, evidence of vertical communication between these two cell layers, across their apical domains, has been uncovered and the PE has been shown to impact aspects of proliferation, patterning, cell fate, and differentiation in the developing retina (reviewed in Atkins and Mardon, 2009; Kumar, 2018). However, these two epithelia are also continuous along a fold called the posterior-lateral margin and the maintenance of this aspect of disc morphology is also critical to produce the perfect crystalline lattice of single eyes essential for fly vision (Weasner and Kumar, 2022). Very little is known about the mechanisms that preserve the overall morphology of the disc, such as those that maintain the superimposition of the PE and retina domains. We show here that AJs in the PE function, via Jub, to regulate Yki activity and epithelial-ECM interactions on the PE side of the disc to maintain this architecture. Disruption of this mechanism ultimately results in malformation of the developing retina – the RDis phenotype – and of the adult compound eye. We were intrigued by this role of AJs in preventing RDis, as we have recently shown that Yki is necessary to prevent RDis in other genetic contexts as well (Neal et al., 2022).

### RDis and Hippo-Yki signaling

In the L3 eye disc, retinogenesis initiates at the posterior of the disc and proceeds anteriorly only in the retinal epithelium (Fig. S2A-A″). Our lab has previously demonstrated that compromised Hippo-Yki signaling can confer retinal fate on presumptive PE cells during L2, leading to the formation of a duplicate retina in the PE (Neal et al., 2020; Zhang et al., 2011). During this PE-to-retina fate change, *de novo* neurogenesis initiates in the presumptive PE at the posterior margin of the disc and progresses anteriorly in a process closely analogous to the normal development of the underlying retinal epithelium (Fig. S2B-B″). The RDis phenotype results from a mechanistically distinct process (Neal et al., 2022), and does not feature ectopic neurogenesis (Fig. S2C-C″).

In the present manuscript, we show that compromising AJs, specifically in the PE, also results in RDis (Fig. 1). In *odd>arm^RNAi^* discs, all of the photoreceptor neurons found in the PE are at a more advanced stage of development, and our observations indicate that the posterior portion of the developing retinal field slides progressively onto the PE plane while neurogenesis continues normally within the retinal epithelium (Fig. 2, Fig. S1). Loss of Arm (β-Cat), α-Cat or DE-Cad in the PE disrupts AJs and leads to RDis, whereas restoring AJs in an Arm-independent manner in *arm^RNAi^* PE is sufficient to suppress this phenotype (Fig. 3). Consistent with the established role of the α-Cat associated Jub protein in positively regulating Yki activity (Rauskolb et al., 2014; Das Thakur et al., 2010), loss of Jub results in RDis (Fig. 4A-A‴), and Yki expression can itself suppress the RDis phenotype induced by *arm* loss-of-function (Fig. 4B-D). Thus, our analyses of the AJ-induced RDis phenotype have returned our focus to the misregulation of Yki.

As part of our prior study of RDis, we obtained two striking results. First, we identified a narrow developmental window, from mid to mid-late L2, when the response of the PE to the loss of Yki changed (Neal et al., 2022). Second, we showed that Protein Phosphatase 2A (PP2A) holoenzymes containing the B′ regulatory subunit Widerborst (Wdb) function only in RDis, and not in PE fate (Neal et al., 2022). While the window of activity of the AJ-Jub-Yki axis is congruent with the RDis phenocritical period identified for loss of PP2A or Yki activity, this raises interesting questions about what drives the transition from control of fate to control of disc morphology. Furthermore, the identification of AJ-Jub as a regulatory hub of Yki activity in RDis raises the issue of its potential role in PE fate. Preliminary experiments using the early PE-specific driver c311-GAL4 suggest that AJs are not required for PE fate (not shown), but further investigation is warranted for this result to be definitive.

That Hpo-Yki signaling relies on distinct regulatory mechanisms to modulate pathway activity over the course of development, particularly when and where differential responses are expected, has been recently shown in a few other contexts. These include the control of cell proliferation by the AJ-Jub-Wts-Yki regulatory axis in different regions of the wing disc (Pan et al., 2018), and the expression of Rhodopsin 5 in a subtype of the R8 photoreceptor which requires Yki but not Jub function (Pojer et al., 2021). Selective utilization of regulatory modules has also been observed in mammalian cell culture, where Ajuba proteins are necessary to regulate proliferation in some contexts, but not in others (Ibar et al., 2018; Jagannathan et al., 2016).

There are undoubtedly many more context-specific regulators of Yki activity that remain to be discovered. Having defined two phenocritical periods for Yki activity in the PE during eye disc development, we believe the PE fate and RDis paradigms can be exploited to understand how pathway output is switched and to identify molecular regulators associated with this process.

### RDis and epithelial-ECM interactions

We also investigated the cellular mechanism that causes RDis, showing that PE cell number is not a determining factor; in fact, cell proliferation and cell death, which are known to be regulated by Yki, are largely unaffected in the eye disc prior to and during the onset of RDis (Fig. 5). Nor do we believe that RDis reflects the re-specification of a boundary, as has been observed in the wing disc when overexpression of constitutively active Yki, but not wt Yki, result in evidence of *de novo* anterior-posterior compartments boundary formation (Bairzin et al., 2020). Specifically, our experiments with PE-expressed Collagen IV-GFP clearly show that the PE-retinal transition is at the correct site along the posterior margin of *odd-Gal4 UAS-arm^RNAi^* eye disc for the first ∼14 h of neurogenesis. Only after this point does it shift anteriorly over the Collagen IV-GFP marked ECM, as the posterior retinal epithelium slides around the posterior fold and onto the PE plane of the disc (Fig. 6A-B′). Thus, we conclude that RDis results from a failure to maintain proper disc morphology rather than aberrant specification of the PE-retina margin.

What then are the tissue-level mechanism(s) by which RDis manifests? Since the L3 eye disc is a rapidly growing tissue, the failure of the PE to remain anchored could result in the retinal domain pushing on the PE tissue and compressing it. Alternatively, the loss of attachment of the PE to its basement membrane could result in a failure to mitigate PE cell contractility and cause the PE to exercise a pulling force on the developing retina. We favor the latter hypothesis, based on the evidence presented in Fig. 6, and summarized below. First, we showed that loss of the βPS-integrin Mys from the PE is sufficient to cause RDis, while restoring βPS-integrin expression in Arm-deficient PE is sufficient to partially suppress the RDis phenotype in that context. Thus, βPS-integrin functions in the control of eye disc morphology, but this does not refute either the “push” or “pull” models. Second, we showed that knocking down Dys, the critical DGC component that connects Dg to the cytoskeleton, reduces the penetrance of RDis in Arm knockdown discs. Reasoning that this reflected the dispersion of tension, we proceeded to knockdown Rok and thereby achieved complete suppression of the RDis phenotype. These results favor the “pull” hypothesis, whereby contractility in the PE leads to RDis.

Whatever the mechanism at the tissue level, how is loss of Yki activity linked to compromised epithelium-ECM interaction? For instance, Yki may be required to promote the expression of critical ECM components or transmembrane connectors in the first place. This control may also extend to intracellular cytoskeletal components. Indeed, Yki is necessary to promote the proper scaling of apical actin stress fiber formation, relative to cell size (Lopez-Gay et al., 2020). In the absence of this mechanism, cells respond inappropriately to morphogenetic mechanical stresses (Lopez-Gay et al., 2020). Conversely, the Hippo cascade is a well know transducer of mechanical forces, and reduced Yki activity may be a downstream effect of compromised epithelial anchoring. In the wing disc, mechanical strain promotes cell flattening that effectively reduces the local concentration of apical transmembrane Hpo activators, and reduces Hpo homodimerization, resulting in increased Yki activity (Fletcher et al., 2018), an outcome that presumes strong epithelium-ECM connections. However, wing PE cells fail to stretch appropriately when Yki is knocked down (Fletcher et al., 2018), suggesting the presence of a feedback loop.

Additional complexity in the regulatory mechanisms are highlighted in recent studies showing that DGCs, like AJs, may also act as Hippo signaling hubs. In fly and vertebrate cardiac tissue, Yki/YAP associates with DGCs (Morikawa et al., 2017; Yatsenko et al., 2020). Furthermore, Yki associated with these complexes is primarily in its phosphorylated and inactive form, suggesting that, like AJs, DGCs serve to promote Hippo activity. In the case of AJ-disrupted RDis discs, where Jub is unable to sequester Wts, Yki phosphorylation is likely increased and this may result in its sequestration at DGCs. Interestingly, the association between DGCs and Yki/Yap requires Dystrophin/DMD (Morikawa et al., 2017; Yatsenko et al., 2020). This suggests an alternative mechanism by which the knockdown of *Dys* rescues RDis. Instead of physically relieving cell tension, it may prevent the sequestration of phosphorylated-Yki at DGCs and permit some level of Yki reactivation in Arm-deficient discs. Thus, a much more detailed analysis will be required to reveal the sequence of events, their interdependence, and their ability to regulate or be regulated by Yki, with respect to the roles of intracellular and ECM factors in governing tissue morphology in the eye disc.

Nonetheless, the general amenability of the *Drosophila* eye disc to genetic, cell biological and developmental analyses make it an exceptional *in vivo* model in which to study complex processes. Our current and recently published data highlight numerous intertwined avenues by which proper organ development is guided by Yki activity, and the RDis paradigm will undoubtedly be useful to further explore the nuances of the regulatory networks involved.

## MATERIALS AND METHODS

### Drosophila genetics and fly lines

See Table S1 for precise genotypes and experimental conditions for all crosses, and Table S2 for a list of stocks used and their sources. The *odd-GAL4* reagent was generated by Judith Lengyel by *p*-element exchange in the *odd^rk111^* enhancer-trap line (Hao et al., 2003). To our knowledge, despite having similar expression characteristics (Levy and Larsen, 2013), this reagent is distinct from that used elsewhere, e.g. (Bras-Pereira et al., 2006). The RNAi-resistant *armadillo* transgenic line was generated by site-directed insertion of a modified plasmid (details below) at attP 86Fb (BestGene).

For temperature shift experiments, flies were crossed in bottles for 3 days at 25°C (∼150 flies/bottle) then transferred to three replicate cages with grape juice plates and fresh yeast paste and cleared for 2 h before embryo collections. Replicate plates were collected every hour and transferred immediately to 18°C. Two of the three plates containing larvae were shifted to 30°C at the indicated times; larvae from the third plate were dissected at time of shift and stained for Elav in order to determine their stage of development (*n*≥10 larvae/experiment). Ommatidial clusters in the L3 eye disc are typically generated at a rate of one row every 2 h (Campos-Ortega, 1980). Experimental larvae were dissected at the late L3 stage. Two control crosses – one at 18°C and one at 30°C – held at constant temperatures for the duration of these experiments were used to ensure that the GAL80^ts^ was functional (active at 18°C and inactive at 30°C), as well as to observe the relative rates of development of experimental larvae. Two independent experiments were conducted.

To assess the delay in Arm depletion following the onset of RNAi expression, bottles were crossed at 18°C until larvae reached the early-mid L3 stage when bottles were then shifted to 30°C. Larvae were dissected at multiple timepoints within the next 24 h, stained for Arm, and analyzed by confocal microscopy. Arm expression in the retina served as an internal control for presence/absence of Arm in the PE. These data revealed that Arm was visibly depleted from presumptive AJs in the PE between 16-24 h after the onset of RNAi expression (at 30°C). We thus inferred the developmental stage at which Arm was lost from the PE based on this delay, the identified stage at the time of dissection, and the above observations of the course of development in experimental flies maintained at 18°C.

### Cloning of RNAi-resistant arm transgenes

Plasmids containing Myc-tagged full-length *arm* cDNA and *arm*_dm (Arm^AJ^) cDNA (Valenta et al., 2011) were obtained from K. Basler (University of Zurich). Codons for amino acids 26-317 were substituted to generate maximal mismatches with the KK102545 RNAi reagent. Both pT2-attB(+)-based plasmids contain the *tubulinα1* promoter and 3′UTR. *Arm^AJ^* encodes amino acids 1-691 and has a single point mutation (D172A).

### Immunohistochemistry

L3 eye discs were dissected in PBS and fixed in 3% paraformaldehyde/phospho-lysine buffer for 30 min, followed by three washes each of 1x PBS and 1x PBS-Triton-X-100 (PBST). Tissue was blocked for 30 min in 5% normal goat serum/PBST, and primary antibodies were incubated overnight in fresh blocking solution. Following primary incubation, tissue was washed three times each in PBST and blocking solution, and secondary antibodies were incubated in fresh blocking solution for at least 4 h. Tissue was then washed three times each in PBST and PBS before mounting. Primary antibodies were from the Developmental Studies Hybridoma Bank [1:200 rat α-Elav (clone 7E8A10, deposited by G.M. Rubin), 1:200 mouse α-Elav (clone 9F8A9, deposited by G.M. Rubin), 1:200 mouse α-Dlg (clone 4F3, deposited by C. Goodman), 1:200 mouse α-Arm (clone N2 7A1, deposited by E. Wieschaus), 1:200 mouse α-Chp (clone 24B10, deposited by S. Benzer and N. Colley), 1:50 rat α-DE-Cad (clone DCAD2, deposited by T. Uemura), 1:100 rat α-α-Cat (clone DCAT1, deposited by M. Takeichi)], Cell Signaling [1:200 rabbit α-Myc (catalog #mAb 2278), 1:1000 rabbit α-HA (catalog #mAb 3724), 1:1000 mouse α-pH3 (catalog #mAb 9706), 1:100 rabbit α-Dcp-1 (catalog #mAb 9578)], or Invitrogen [1:10^4^ rabbit α-GFP (catalog #A-6455)]. Cy2-, Cy3-, and Cy5-conjugated goat α-mouse, rat or rabbit secondary antibodies were used at 1:400 (Jackson ImmunoResearch). Hoechst 33342 (1:10^5^; Invitrogen) was included in either the secondary antibody solution or in the mounting medium as counterstain, where indicated. Discs were mounted in medium consisting of 65% glycerol and 2.5% *n*- propyl gallate (Sigma) in 1xPBS, or Vectashield (Vector Labs).

### Image acquisition and processing

Confocal stacks were recorded in Leica LASX software using a Leica DM5500Q microscope with SPEII confocal head. Images were processed in LASX and in Adobe Photoshop; only global manipulations were applied to images. Fluorescence intensities were adjusted both pre- and post-imaging for best presentation; no quantitative inferences should be made based on fluorescence intensity. Figures were assembled in Adobe Illustrator.

### Cell counting

For data presented in Fig. 5, discs were stained for the sub-apical marker Dlg and/or anti-pH3 and/or anti-Dcp-1, and cells were counted throughout the PE, using the FIJI Cell Counter plugin, by two independent observers who were blinded to the genotypes.

### Reproducibility

Representative images are shown in all figures. All experiments were repeated at least once. Given the delayed manifestation of the RDis phenotype (Fig. S1), discs were counted as ‘rescued’ only if they had at least 15 rows of Elav-positive photoreceptor clusters, such that RDis would be fully penetrant in equivalent ‘non-rescued’ discs. Precise numbers of discs observed are given in the results and relevant figure captions.

## Supplementary Material

10.1242/biolopen.059579_sup1 Supplementary information
