# Supplementary material for: Peripodial adherens junctions regulate Ajuba-Yorkie signaling to preserve fly eye morphology
Source: Biol Open. 2023 Mar 27;12(3):bio059579. doi: 10.1242/bio.059579 (PMC10084860; doi:10.1242/bio.059579)
Supplement: Supplementary information [file biolopen-12-059579-s1.pdf]

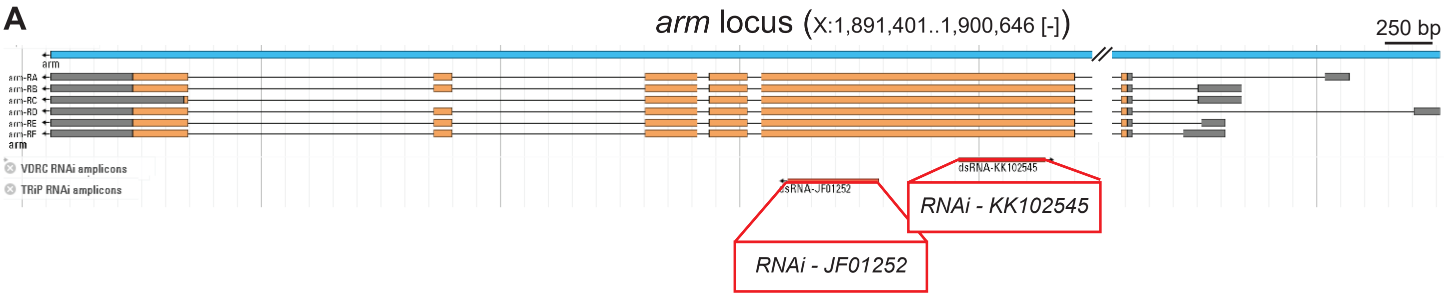

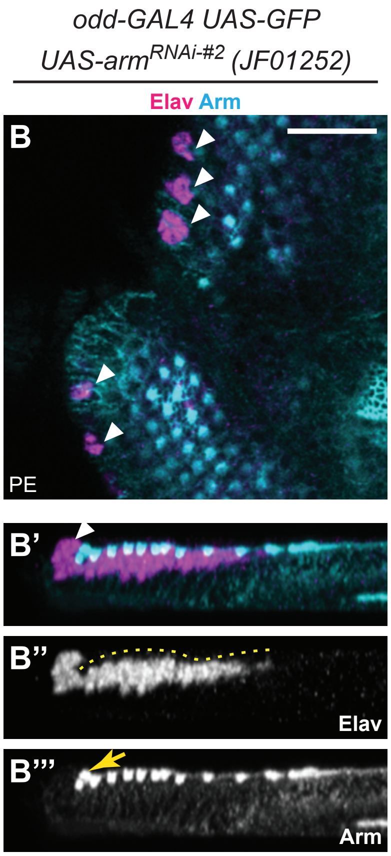

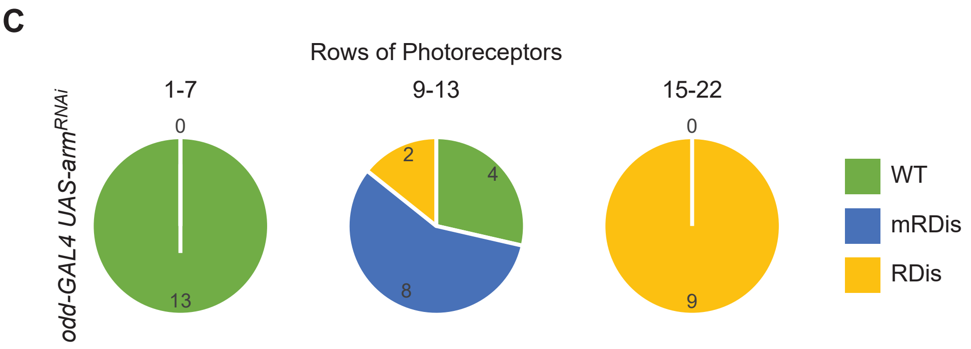

D

# Rows	# Discs	Phenotype WT	mRDis	RDis
1	3	3	0	0
2	1	1	0	0
3	0		(n/a)	
4	3	3	0	0
5	3	3	0	0
6	2	2	0	0
7	1	1	0	0
8	0		(n/a)	
9	3	1	2	0
10	1	1	0	0
11	4	0	3	1
12	3	1	2	0
13	3	1	1	1
14	0		(n/a)	
15	3	0	0	3
16	1	0	0	1
17	0		(n/a)	
18	1	0	0	1
19	1	0	0	1
20	0		(n/a)	
21	1	0	0	1
22	2	0	0	2

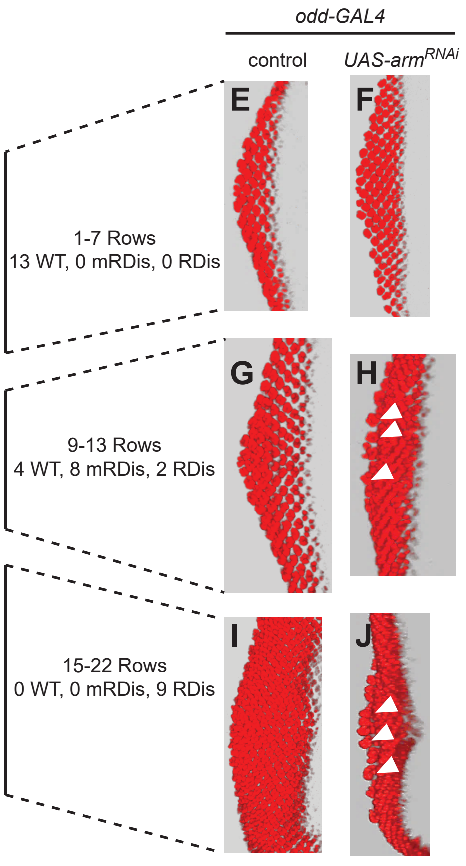

Fig. S1. Validation of the *arm*-mutant phenotype and developmental progression of RDis. (**A**) JBrowse view, adapted from FlyBase, of the *arm* locus. The regions of the **gene targeted by the two transgenic RNAi stocks used in this manuscript are indicated.** The VDRC KK102545 reagent (*arm*^{RNAi}) was used throughout the primary figures in the manuscript, while the TRiP JF01252 reagent (*arm*^{RNAi} #2) was used only to confirm the mutant phenotype, as shown below). (**B-B'''**) Expression of JF01252 in the PE using *odd-GAL4* resulted in the presumptive RDis phenotype (n=56, 88% penetrance; scale bar = 25 µm). Mislocalized Elav-positive (magenta) cell clusters (white arrowheads) were observed in the PE plane (B-B'') and Arm immunoreactivity (cyan) was absent from the PE layer (B-B', B'''). The disc lumen is indicated by a dashed yellow line. (B - limited Z-projection of X-Y optical sections; B'-B'''' - limited Y-projection of X-Z optical sections). (**C**) Plots illustrating the relative penetrance of retinal curling in mild (blue) or more severe (yellow) RDis phenotypes, versus wild-type development (green). Phenotypes are shown as a function of retinal developmental state, measured as the number of rows of Elav-positive cell clusters in the disc. (**D**) Observations compiled in order to generate plots in (A). (**E-J**) 3D reconstructions of Elav-positive cells (red) from discs of the indicated genotypes at the indicated developmental stages. Mislocalized Elav-positive cell clusters are indicated by white arrowheads.

Biology Open • Supplementary information

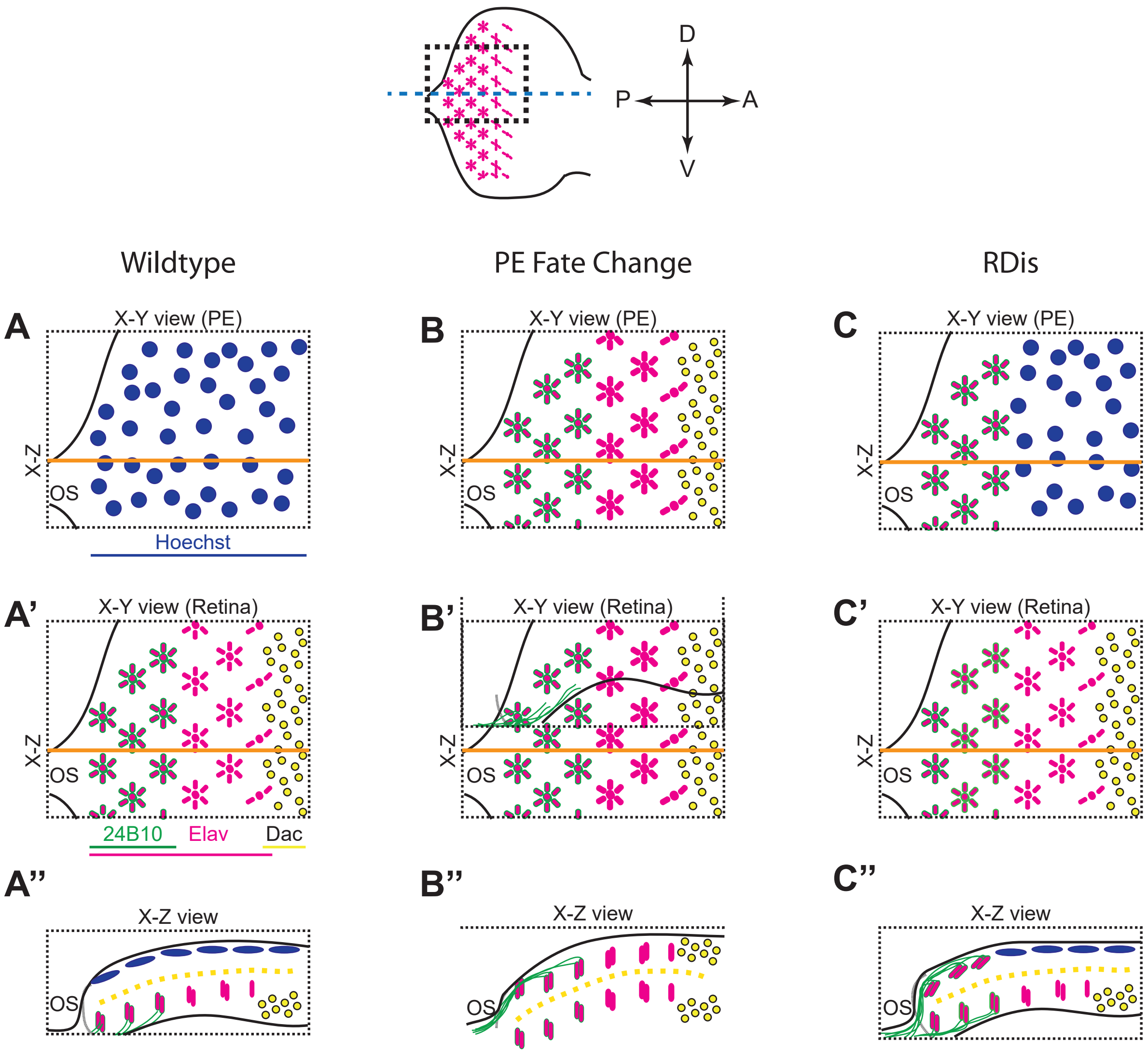

Biology Open • Supplementary information

Fig. S2. Schematics of wildtype, PE fate-converted and RDis eye discs. (**A-C''**) Schematics illustrating wild-type (WT) disc patterning (A-A''), and patterning in discs with PE-to-retina fate change (B-B'') and RDis (C-C''), adapted from (Neal et al., 2020, Neal et al., 2022). Patterns of retinal markers (Elav – all neurons, magenta; Dac – neural progenitors, yellow circles; the 24B10 antigen (Chaoptin) – maturing photoreceptor neurons, green outlines and axons) and cells of the peripodial epithelium (PE, large blue circles/ovals) within late 3rd instar *Drosophila* eye imaginal discs are illustrated (OS – optic stalk). Orange lines in the X-Y views (A-A', B-B', C-C') indicate the plane of the orthogonal (X-Z) view shown below (A'', B'', C'', respectively). The lumen between the epithelial layers is indicated by a dashed yellow line in X-Z views. In wildtype discs, retinogenesis is initiated at the posterior margin and proceeds anteriorly only in the retinal epithelium. In contrast, a PE fate change is marked by a second independent wave of neurogenesis in the presumptive PE domain. In RDis discs, a portion of the posterior retinal tissue, marked by 24B10-positive neurons, becomes mislocalized onto the PE side of the disc, but ectopic retinogenesis is not detected.

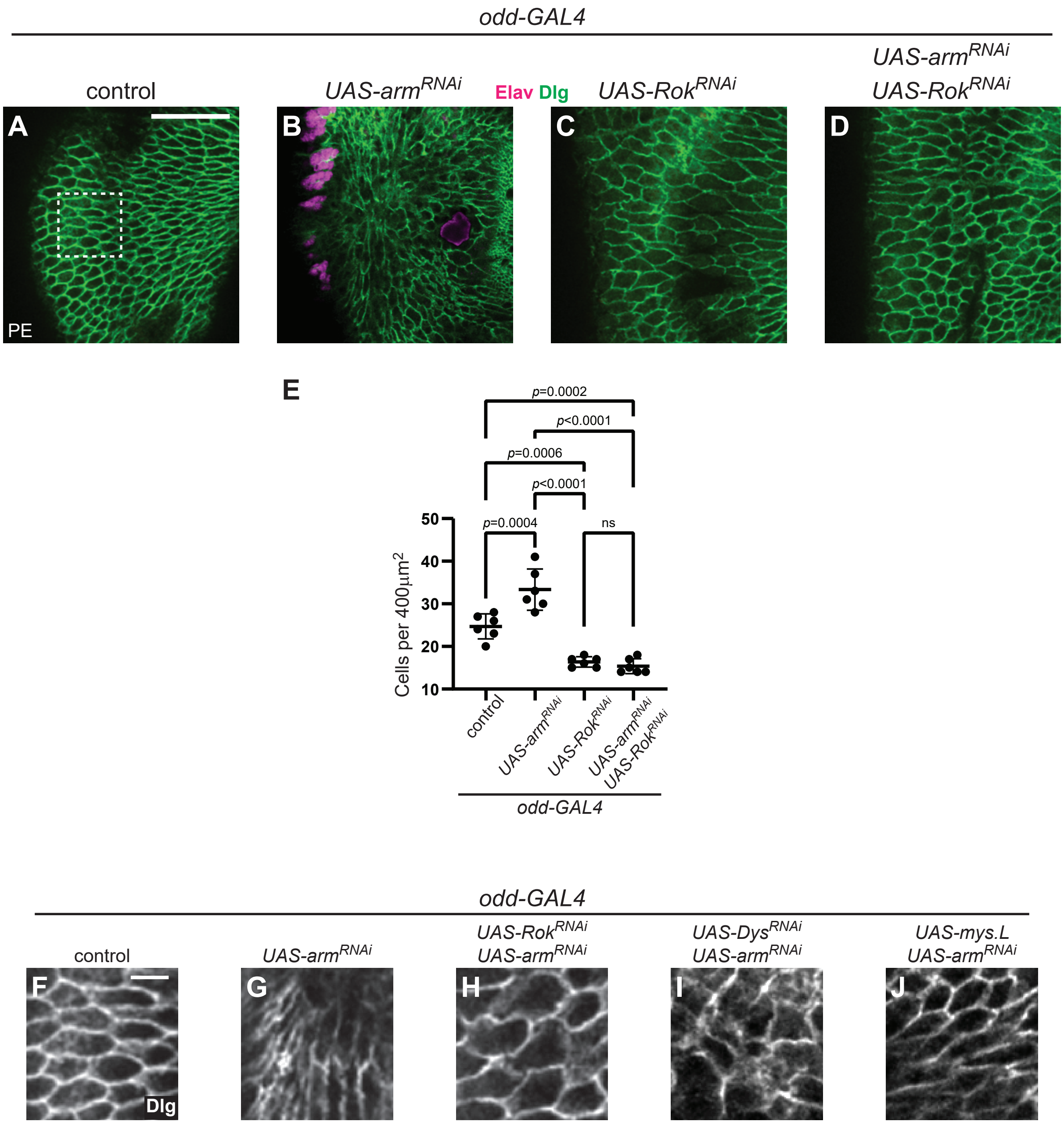
odd-GAL4
control
UAS-arm^{RNAi}
Elav Dlg
UAS-Rok^{RNAi}
UAS-arm^{RNAi}
UAS-Rok^{RNAi}
A
B
C
D
PE
E
p=0.0002
p<0.0001
p=0.0006
p<0.0001
p=0.0004
ns
Cells per 400μm^2
50
40
30
20
10
control
UAS-arm^{RNAi}
UAS-Rok^{RNAi}
UAS-arm^{RNAi} UAS-Rok^{RNAi}
odd-GAL4
odd-GAL4
control
UAS-arm^{RNAi}
UAS-Rok^{RNAi} UAS-arm^{RNAi}
UAS-Dys^{RNAi} UAS-arm^{RNAi}
UAS-mys.L UAS-arm^{RNAi}
F
G
H
I
J
Dlg

Fig. S3. PE cell tension may contribute to retinal displacement. (**A-D**) Limited Z projections of the PE planes of discs of the indicated genotypes. Neurons (Elav, magenta) and sub-apical profiles of cells (marked by Dlg, green) are shown. An example 20 µm x 20 µm region of interest (ROI) at the center posterior of the disc, where cell counts were made, is illustrated in A. (**E**) Cells fully or partially within ROIs were manually counted. Individual ROI counts (filled circles) are shown, along with the mean and standard deviation for each genotype, as indicated. Means were compared by one-way ANOVA, with *post hoc* Tukey test for pairwise differences; precise *p* values are shown. ns – not significant. Data analyzed in GraphPad Prism (v. 9.1). (**F-J**) Representative ROIs (as above) of discs of the indicated genotypes; Dlg staining in grayscale. In contrast to wt (F) and RDIs discs (G), the various genetic rescue paradigms (H-J) produced PE cells with different sizes and aspect ratios. A more comprehensive analysis of PE cells in these paradigms is warranted. The scale bars in panel A (25 µm) and F (5 µm) apply to panels A-D, F-J, respectively.

Table S1. Precise genotypes and experimental conditions related to data presented in this work.

Click here to download Table S1

Table S2. Origin and genotypes of transgenes used in this work.

Click here to download Table S2
